# Insights into the actions of angiotensin-1 receptor (AT1R) inverse agonists: Perspectives and implications in COVID-19 treatment

**DOI:** 10.17179/excli2021-3412

**Published:** 2021-02-08

**Authors:** Luana Heimfarth, Mario Adriano dos Santos, José Augusto Barreto-Filho, André Sales Barreto, Fabrício Nunes Macedo, Adriano Antunes de Souza Araújo, Paulo Martins-Filho, Marcus Tullius Scotti, Luciana Scotti, Lucindo José Quintans-Júnior

**Affiliations:** 1 Laboratory of Neuroscience and Pharmacological Assays (LANEF), Department of Physiology, Federal University of Sergipe, São Cristovão, Sergipe, Brazil; 2 Department of Medicine, Federal University of Sergipe, Aracaju, Sergipe, Brazil; 3 Postgraduate Program in Health Sciences, Federal University of Sergipe, Aracaju, Sergipe, Brazil; 4 Laboratory of Cardiovascular Pharmacology, Department of Physiology, Federal University of Sergipe, Sao Cristovão, Sergipe, Brazil; 5 Faculdade Estácio de Sergipe, Aracaju, Sergipe, Brazil; 6 Department of Pharmacy, Federal University of Sergipe, Sao Cristovão, Sergipe, Brazil; 7 Cheminformatics Laboratory- Postgraduate Program in Natural Products and Synthetic Bioactive, Federal University of Paraiba-Campus I, 58051-970, João Pessoa, PB, Brazil

**Keywords:** SARS-CoV-2, angiotensin-converting enzyme, drugs, renin-angiotensin-aldosterone system

## Abstract

New coronavirus SARS-CoV-2 (COVID-19) has caused chaos in health care systems. Clinical manifestations of COVID-19 are variable, with a complex pathophysiology and as yet no specific treatment. It has been suggested that the renin-angiotensin-aldosterone system has a possible role in the severity of cases and the number of deaths. Our hypothesis is that drugs with inverse agonist effects to the angiotensin-1 receptor can be promising tools in the management of patients with COVID-19, possibly avoiding complications and the poor evolution in some cases. Any risk factors first need to be identified, and the most appropriate time to administer the drugs during the course of the infection also needs to be established. Several angiotensin receptor blockers (ARB) have a favorable profile and are important candidates for the treatment of COVID-19. In this review we discussed a set of compounds with favorable profile for COVID-19 treatment, including azilsartan, candesartan, eprosartan, EXP3174, olmesartan, telmisartan, and valsartan. They are effective as inverse agonists and could reduce the “cytokine storm” and reducing oxidative stress. As COVID-19 disease has several evolution patterns, the effectiveness of ARB therapy would be related to infection "timing", patient risk factors, previous use of ARBs, and the specific molecular effects of an ARB. However, controlled studies are needed to identify whether ARBs are beneficial in the treatment of patients with COVID-19.

## Introduction

Sudden or emerging pathogen outbreaks have always been a challenge to public health worldwide. A recent outbreak of coronavirus, identified as SARS-CoV-2, started in Wuhan, China and quickly spread to almost all other countries. According to the WHO, 76,250,431 cases have been confirmed globally, with 1,699,230 deaths up to December, 22, 2020 (WHO, 2020[128]). Most non-survivors were older (>80 years old) with severe other diseases and more likely to develop acute organ dysfunction (Guo et al., 2021[35]). In addition, extremely low lethality was observed in young patients (Flacco et al., 2020[28]). During the last five months, various studies about coronavirus disease 2019 (COVID-19, the disease caused by the SARS-CoV-2 virus) have described its clinical features, reported laboratory findings and provided diagnostic evaluations of the disease. COVID-19 mortality rates vary by country, and are also influenced by the clinical profile of patients, and the presence of other comorbidities (Carlino et al., 2020[13]; Scholz et al., 2020[103]). No proven effective drug against SARS-CoV-2 has yet been found and treatment is based on symptom management and life support, although some advances have been made with the use of anticoagulants and dexamethasone-type corticosteroid drugs. Thus, the lethality of the disease for patients with comorbidities, older adults and those with secondary infections is still a challenge in those who develop the severe form of COVID-19 (Iaccarino et al., 2020[45]). 

The clinical manifestations of COVID-19 are extremely complex and variable. SARS-CoV-2 infection may be asymptomatic or cause mild symptoms such as fever, dry cough, shortness of breath, headache, dyspnea, diarrhea and vomiting (Huang et al., 2020[44]). In some patients, the SARS-CoV-2 infection can have a worse prognosis, requiring hospitalization (Bloomgarden 2020[10]; Cossarizza et al., 2020[19]). In severe cases, COVID-19 may progress to acute respiratory distress syndrome (ARDS), followed by refractory metabolic acidosis, coagulation dysfunction, septic shock, multiple organ failure and, consequently, death (She et al., 2020[106]; Wang et al., 2020[124]). Despite the tropism of SARS-CoV-2 for the respiratory airways (Wang et al., 2020[124]), several other organs are also affected by the virus, explaining the cases of multiorgan failure. The cardiovascular and renal systems appear to have complex interactions with COVID-19, making patients more predisposed to severe cardiovascular damage or renal failure (Deep et al., 2020[22]; Tomasoni et al., 2020[120]). Recent retrospective clinical findings have established an association between the incidence of vascular thrombosis or thromboembolic events and COVID-19 severity (Huang et al., 2020[44]; Tang et al., 2020[116]). 

In addition, some biological mechanisms that occur during SARS-CoV-2 infection are thought to be pathogenic and could be associated with poor prognosis of the disease. With disease progression, infiltration of neutrophils, macrophages, and red blood cells (Matthay et al., 2019[71]) and the accumulation of protein-rich edema in the alveoli occurs, causing local production of pro-inflammatory cytokines associated with adaptive and innate immune cells. These factors contribute to the exaggerated recruitment of inflammatory cells, protease release, cytokine production and oxidative stress, that are responsible for the disruption of the blood-alveolar barrier, intrapulmonary hemorrhage, pulmonary edema, and the dangerous impairment of gas exchange (Channappanavar and Perlman 2017[14]), provoking massive injuries in lung microvascular endothelial and epithelial cells (Matthay et al., 2019[71]). This excessive uncontrolled inflammatory response seems to be related to ARDS, multiorgan failure (Inciardi et al., 2020[50]) and even the death of patients with COVID-19.

The role of the renin-angiotensin-aldosterone system (RAS), particularly the role of ACE2 (angiotensin-converting enzyme 2) receptors, has been studied in SARS-CoV infections and appears to be associated with the progression of COVID-19 (McMillan and Uhal 2020[73]; Ni et al., 2020[85]). ACE2 is an enzyme that catalyzes the conversion of angiotensin I to angiotensin (1-9) and angiotensin II to angiotensin (1-7) and plays a key role in the renin-angiotensin-aldosterone system (RAS) (Voors et al., 1998[123]). Ang II, the main active RAS component, acts on angiotensin-II type 1 receptors (AT1R), exerting its physiological (Keidar et al., 2007[53]) and antagonistic effects through the angiotensin-II type 2 receptors (AT2R). SARS-CoV binds to the ACE2 receptor to achieve intracellular invasion and infectivity (Ni et al., 2020[85]; Tang et al., 2020[116]) with the virus using S protein priming by the transmembrane protease serine 2 (TMPRSS2) for cell infection. TMPRSS2 activity is crucial for viral spread and pathogenesis in the infected host and seems to be responsible for the ease of transmissibility of SARS-CoV-2 (Matsumoto et al., 2003[69]; Hoffmann et al., 2020[43]). 

ACE2 is also highly expressed in the kidneys, heart, respiratory tract, and gastrointestinal tract tissues, with lower expression levels in the brain and blood cells (Hamming et al., 2004[39]; Gkogkou et al., 2020[31]). The expression of ACE2 is responsible for the susceptibility of human cells to SARS-CoV-2 (Hardenberg and Luft 2020[40]). In addition, ACE2 is expressed at the same sites where pro-inflammatory cytokines released following SARS-CoV infection are produced (He et al., 2006[41]). It is associated with the pulmonary tropism of the viruses found in diseases caused by other similar coronaviruses (Imai et al., 2005[48]). Thus, the modulation of ACE2 activity and its secondary effects may exert a protective role against cardiac and/or lung injury, ARDS and other COVID-19 complications. However, the correlations between the different stages of SARS-CoV-2 infection and the RAS system remain to be clarified (Vitiello and Ferrara, 2020[122]).

The detrimental actions of the Ang II AT1R-mediated inflammatory response have been demonstrated in various models of ARDS SARS-CoV-induced acute respiratory failure (Imai et al., 2005[48]; Kuba et al., 2005[56]). Moreover, some studies have shown that experimentally ARDS and lung fibrosis can be prevented by administration of Angiotensin-II receptor antagonists (ARBs), limiting pulmonary disease progression (Wösten-van Asperen et al., 2011[131]). This implies that ARBs could act in SARS-CoV-2 infection, modifying disease progression (Dublin et al., 2020[23]). In addition, ARBs possess inverse agonist properties that give them an additional pharmacological effect and improves drug efficacy (Akazawa et al., 2009[2]). Thus, drugs that act on the AT1R have been proposed as a treatment for COVID-19 (Gurwitz, 2020[37]). Interestingly, Zhang et al. (2020[141]) found that among COVID-19 patients hospitalized with hypertension, inpatient treatment with ACEI/ ARB was related to a lower risk of all-cause mortality.

Although the role of the RAS has been extensively studied in COVID-19 patients, there are unquestionable gaps of information in relation to this topic, especially regarding the role of AT1-inverse agonists and their action in SARS-CoV-2 infection. We, therefore, performed a review of the AT1-inverse agonists that could be of benefit in the treatment of patients with severe COVID-19. Based on our examination of basic and clinical studies, we hypothesize that angiotensin-1 receptor (AT1R) inverse agonist drugs may be a promising tool in the management of COVID-19 patients, avoiding complications and the development of the severe phase of the disease, and the subsequent poor outcomes.

## ARBs and Possible Implications for COVID-19

### AT1-inverse agonists in the treatment of COVID-19

**Figure 1:**
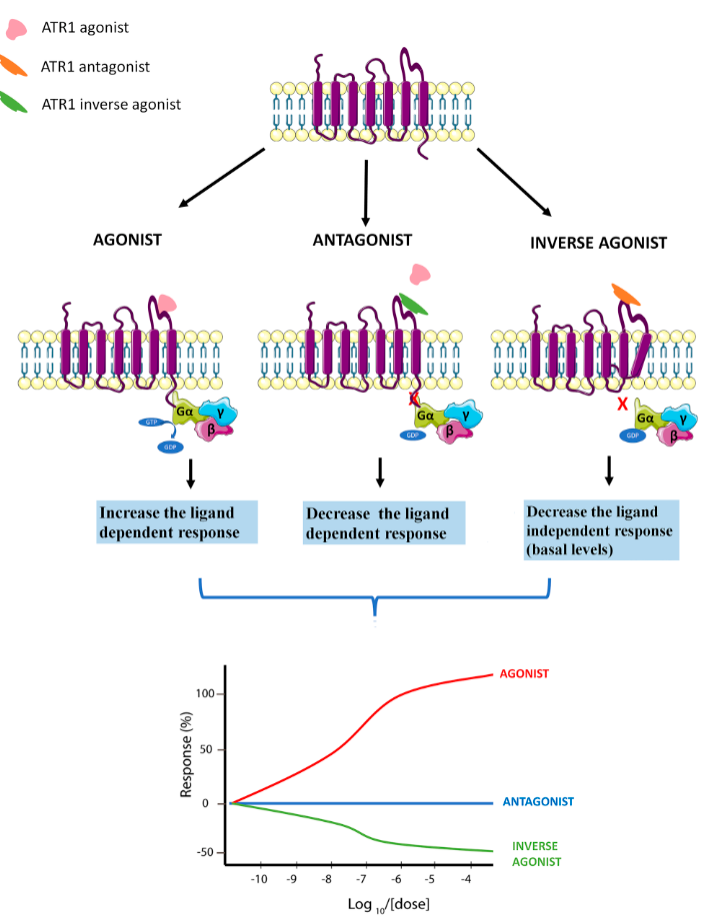
Schematic representation of AT1 agonist, AT1-antagonist and AT1-inverse agonist biological response

An inverse agonist is defined as a ligand that binds to the same receptor as the agonist but induces opposite effects (Akazawa et al., 2009[2]). While the agonist molecule stabilizes receptor conformations that increase signaling through G proteins, the inverse agonists promote other conformations that decrease the basal, agonist-independent level of signaling and modulate the response, besides suppressing stimuli from the agonist (Milligan 2003[75]; Akazawa et al., 2009[2]). The agonist is a molecule that binds and activates a receptor to produce a biological response, whereas the antagonist inhibits the action of the agonist (Figure 1(Fig. 1)).

In this context, we can highlight AT1-inverse agonists that may exhibit enhanced therapeutic effects for various disease states (Takezako et al., 2017[113]). Studies have shown that several G protein-coupled receptors (GPCRs), including the AT1R, show spontaneous activity, even in the absence of an agonist (Akazawa et al., 2009[2]). AT1-inverse agonists regulate several important signaling pathways, leading to anti-inflammatory (Gupta et al., 2020[36]), anti-apoptotic (Kanamori et al., 2007[52]), antioxidant (Occhieppo et al., 2020[87]), and immunomodulatory (Yuan et al., 2016[139]) effects, and consequently opposing the effects of angiotensin II. This evidence supports the potential actions of AT1-inverse agonists in the fight against COVID-19. 

Several ARBs have shown inverse agonist effects and favorable profiles for COVID-19 treatment, including azilsartan (Takezako et al., 2017[113]), candesartan (Miura et al., 2006[76]; Qin et al., 2009[95]), eprosartan (Takezako et al., 2017[113]), EXP3174 (Miura et al., 2006[76]; Qin et al., 2009[95]), olmesartan (Miura et al., 2006[76]; Qin et al., 2009[95]), telmisartan (Takezako et al., 2017[113]), and valsartan (Takezako et al., 2017[113]), with each drug having different features and efficacy as inverse agonists (Takezako et al., 2018[115]). Table 1(Tab. 1) and 2(Tab. 2) summarize the main molecular and physiologic actions of AT1-inverse agonists, and Figure 1(Fig. 1) shows their chemical structure.

#### Alzisartan

Azilsartan is a drug approved worldwide for the treatment of hypertension, either as a prodrug (azilsartan medoxomil) or a primary compound (Pradhan et al., 2019[93]) and has more affinity to the AT1R than to the AT2 receptor. It is one of the most effective approved ARBs tested to date in relation to the reduction of blood pressure (Al-Majed et al., 2020[3]). Azilsartan could provide an effective treatment against COVID-19 by ameliorating the deleterious effects of angiotensin II, such as cardiac hypertrophy, fibrosis, and insulin resistance (Arumugam et al., 2016[6]). In addition, it increases left ventricular diastolic function (Sakamoto et al., 2015[101]), reduces cardiovascular sympathetic activity (Kusuyama et al., 2014[57]), and restores endothelial function (Matsumoto et al., 2003[69]). Therefore, these cardiovascular protective actions could help in the complications that have been found in SARS-CoV-2 patients (Table 1(Tab. 1); References in Table 1: Arumugam et al., 2016[6]; Barone et al., 2001[9]; Chen et al., 2020[15]; De Tommasi et al., 2003[21]; Elliott, 1999[26]; Fan et al., 2016[27]; Furukawa et al., 2009[29]; Galetta et al., 2010[30]; Hikosaka et al., 2002[42]; Ichihara et al., 2006[46]; Iino et al., 2012[47]; Kaliuzhin et al., 2013[51]; Khuman et al., 2016[54]; Kim and Im, 2019[55]; Kusuyama et al., 2014[57]; Kyotani et al., 2010[58]; Lewandowski et al., 2008[61]; Ma et al., 2012[67]; Matsumoto et al., 2014[70]; Matys et al., 2003[72]; Miyoshi et al., 2011[77]; Mizuta et al., 2008[78]; Mojiri-Forushani et al., 2018[80]; Navar et al., 1996[84]; Ojima et al., 2011[89]; Perl et al., 2010[90]; Perrone-Filardi et al., 2009[91]; Poisner et al., 2018[92]; Qin et al., 2009[95]; Quinn Baumann et al., 2013[96]; Remková et al., 2008[98]; Sakamoto et al., 2015[101]; Sharaf El-Din and Abd Allah, 2016[105]; Soliman, 2014[108]; Sukumaran et al., 2011[111]; Takezako et al., 2018[115]; Tanno et al., 2016[117]; Timmermans, 1999[119]; Tomasoni et al., 2020[120]; Wong et al., 1996[130]; Wu et al., 2015[132]; Yin et al., 2012[135]; Yokoyama et al., 2005[136]).

Moreover, the modulation of Ang II could be a key element in the immunomodulatory and anti-inflammatory effects of azilsartan, making it an attractive candidate for mitigating the inflammatory condition observed in SARS-CoV-2 patients. This drug attenuates the release of IL1-β, TNF-α and IL-6 pro-inflammatory cytokines, as well as increasing the production of the important anti-inflammatory molecule IL-10. Azilsartan also downregulates ROS formation, protecting the tissue from oxidative damage (Liu et al., 2016[66]; Gupta et al., 2020[36]), and it is able to inhibit the apoptotic pathway by blocking caspase activation (Gupta et al., 2020[36]) (Table 2(Tab. 2); References in Table 2: Ahmed and Mohamed, 2019[1]; AlSaad et al., 2020[4]; Araujo et al., 2018[5]; Aziz et al., 2020[8]; Chen et al., 2018[16]; Goel et al., 2018[32]; Gong et al., 2019[33]; Graus-Nunes et al., 2019[34]; Gupta et al., 2020[36]; Haas et al., 2019[38]; Imran et al., 2019[49]; Kanamori et al., 2007[52]; Kim and Im, 2019[55]; Labiós et al., 2008[59]; Lakshmanan et al., 2012[60]; Li et al., 2016[62]; Lin et al., 2014[64]; Lin et al., 2015[65]; Liu et al., 2016[66]; Matsumoto et al., 2003[69]; Mohany et al., 2020[79]; Morsy et al., 2015[81]; Mukaddam-Daher et al., 2009[82]; Nakamura et al., 2013[83]; Occhieppo et al., 2020[87]; Ohshima et al., 2014[88]; Prasad, 2006[94]; Qin et al., 2009[95]; Rashikh et al., 2014[97]; Saber et al., 2019[99][100]; Sakr et al., 2016[102]; Shaaban et al., 2014[104]; Sharaf El-Din and Abd Allah, 2016[105]; Takeuchi et al., 2013[112]; Tanno et al., 2016[117]; Wang et al., 2019[125]; Wu et al., 2015[132]; Xin et al., 2020[133]; Zhang et al., 2019[142]). Azilsartan, therefore, presents anti-inflammatory, antioxidant and anti-apoptotic profiles that could contribute to the suppression of multiorgan failure, and disseminated intravascular coagulation, reducing the severity of SARS-CoV-2 infection.

#### Candesartan

Candesartan is an oral selective AT1R blocker available as a pro-drug, candesartan cilexetil, which undergoes hydrolysis in the gastrointestinal tract during absorption to its active form. Candesartan also acts as an inverse agonist, decreasing the basal activity of the AT1R. Accumulating evidence has demonstrated that candesartan could be a very attractive candidate for COVID-19 treatment due to its anti-inflammatory (Yu et al., 2019[138]), 2019), anti-apoptotic (Goel et al., 2018[32]) and antioxidant (Occhieppo et al., 2020[87]) activities. 

Zhang et al. (2020[141]) and Khuman et al. (2016[54]) showed that candesartan protects the cardiovascular system from stroke, myocardial infarction, atherosclerosis, and hypertension by modulating vascular remodeling, thereby preventing the vascular damage-induced by SARS-CoV-2. Furthermore, intermediate to high doses of candesartan ameliorate the progression of nephropathy, showing renoprotective properties (Callera et al., 2016[12]). According to Kim and Im (2019[55]) candesartan ameliorates pulmonary injury by attenuating eosinophil infiltration and inhibiting mucin production in the lung after damage. 

In addition, candesartan modulates several inflammatory pathways (Gong et al., 2019[33]; Kim and Im, 2019[55]) and could reduce the cytokine storm induced by SARS-CoV-2 infection. Kim and Im (2019[55]) and Gong et al. (2019[33]) reported that candesartan attenuates IL1-β, IL-2, IL-4, IL-5, IL-6, IL13 and IFN-γ production and upregulates IL-10 secretion. Moreover, this ARB could block NKκB (Ahmed and Mohamed, 2019[1]) and COX-2 activation, as well as inhibit iNOS (Gong et al., 2019[33]), decreasing the production of inflammatory mediators. 

Candesartan could also mitigate oxidative imbalance by reducing ROS formation (Occhieppo et al., 2020[87]), attenuating oxidative stress, and restoring cellular homeostasis. The reduction in ROS generation causes a decrease in lipid oxidation and, consequently, protects cell membrane integrity and ion transportation across them. Moreover, candesartan inhibits the apoptotic pathway (Goel et al., 2018[32]) and MAPK activation (Ahmed and Mohamed, 2019[1]), attenuating cell death and cellular homeostasis disruption. Thus, the maintenance of oxidative and cellular homeostasis, as well as the reduction in inflammatory process, induced by candesartan could be associated with a reduction in the severity of COVID-19. 

#### Eprosartan

Eprosartan is a nonbiphenyl nontetrazole AT1R blocker routinely used in the treatment of hypertension. Although various ARBs have inverse agonism, eprosartan could be considered a better therapeutic option than other ARBs for the treatment of diseases due to its capacity to act in the active state of the AT1R (Takezako et al., 2018[115]). Studies have shown that eprosartan may protect the cardiovascular, renal and pulmonary systems from inflammatory (Mukaddam-Daher et al., 2009[82]), oxidative (Morsy et al., 2015[81]) and apoptotic (Mukaddam-Daher et al., 2009[82]) damage. Barone et al. (2001[9]) reported that eprosartan reduces cardiac hypertrophy and heart failure and preserves cardiac and renal structural integrity and maintains normal function of the heart and kidneys. Furthermore, this ARB significantly decreased platelet activation and endothelial dysfunction in patients, protecting against organ failure.

According to Mukaddam-Daher et al. (2009[82]) and Labiós et al. (2008[59]), eprosartan mitigates inflammatory and oxidative conditions, respectively. This AT1R blocker reduces the production of the inflammatory mediators IL1-β, TNF-α (Mukaddam-Daher et al., 2009[82]) and oxide nitric (Morsy et al., 2015[81]). Furthermore, eprosartan decreases ROS formation by upregulating enzymatic and non-enzymatic antioxidant defenses, attenuating tissue lipoperoxidation and protecting the cell from apoptosis (Mukaddam-Daher et al., 2009[82]; Morsy et al., 2015[81]). Therefore, eprosartan could be suggested for the treatment of COVID-19 patients due to its capacity to mitigate the “cytokine storm” by modulating the production of cytokines, mainly IL-6, and reducing oxidative stress. Increased cytokine levels have been closely correlated with ARDS severity and a worse prognosis in COVID-19.

#### EXP3174

Losartan is metabolized by cytochrome P450 enzymes to active 5-carboxylic acid derivative EXP3174. This compound is an active metabolite of losartan, with a higher affinity to AT1 and a longer half-life than losartan (Stearns et al., 1995[110]; Wong et al., 1996[130]). Thus, the pharmacological activities of losartan are predominantly mediated by EXP3174 (Wong et al., 1996[130]).

Similar to other ARBs, losartan and its active metabolite could reduce organ failure, and protect tissues from damage caused by SARS-CoV-2-infection (AlSaad et al., 2020[4]; Xin et al., 2020[133]). It has also been found that EXP3174 plays a role in preventing heart failure, as well as reducing hypertension (Timmermans, 1999[119]). In addition, losartan and EXP3174 have been reported to inhibit platelet aggregation, showing antithrombotic activity (Matys et al., 2003[72]). According to Poisner et al. (2018[92]) losartan, and probably its metabolite EXP3174, prevented vasculitis and inflammatory processes in the lung, showing that these compounds could protect against acute respiratory distress syndrome and severe COVID-19.

Additionally, EXP3174 modulates IL-6, IL-1β, TNF-α, IFN-γ and TGF-β secretion (Lin et al., 2014[64]; Wang et al., 2019[125]; AlSaad et al., 2020[4]), and downregulates MAPK and NFκB pathway activation (Wang et al., 2019[125]). Moreover, losartan and EXP3174 were able to ameliorate organ injury by increasing catalase, glutathione peroxidase and superoxide dismutase activity, and consequently decreasing ROS generation and oxidative tissue damage (Lin et al., 2014[64]; AlSaad et al., 2020[4]). According to Xin et al. (2020[133]) losartan can also prevent organ failure by inhibiting the activation of the intrinsic apoptotic cascade, thereby reducing cell death. Therefore, Exp3174 could be an important candidate in the management of COVID-19 patients due to its capacity to block the fulminant response by the immune system and suppress multiple organ failure, contributing to a reduction in the severity of cases.

#### Olmesartan

Olmesartan is a 3^rd^ generation angiotensin-II receptor blocker approved by the FDA for the treatment of mild to severe hypertension. It has been shown that olmesartan prevents ventricular hypertrophy and fibrosis in hypertensive rats with advanced heart failure (Yoshida et al., 2004[137]), and inhibits vascular remodeling, immune cell infiltration, and endothelial dysfunction, attenuating the severity of the heart injury (Chen et al., 2020[15]). Moreover, studies have reported that olmesartan exerts renoprotective effects (Si et al., 2014[107]) and ameliorates lung damage by the modulation of the pro-fibrogenic cytokine TGF-β1, and by its antioxidant effect (Sharaf El-Din and Abd Allah, 2016[105]).

In addition, olmesartan might also be a promising therapeutic approach in SARS-CoV-2 infection due to its capacity to modulate ACE2 expression (Araújo et al., 2018[5]) and to regulate inflammatory, oxidative and apoptotic signaling pathways (Kanamori et al., 2007[52]; Lakshmanan et al., 2012[60]; Saber et al., 2019[100]), leading to a reduction in multiorgan failure. Studies suggest that olmesartan can mitigate the “cytokine storm” since this drug decreases the production of IL1-β and TNF-α, and particularly IL-6 and TGF-β1. It also elevates anti-inflammatory cytokine IL-10 levels (Sharaf El-Din and Abd Allah, 2016[105]; Saber et al., 2019[100]). In fact, this ARB inhibits important inflammatory molecules that play a pivotal role in the inflammatory cascade, including the MAPK family and the transcriptional factor NFκB (Lakshmanan et al., 2012[60]; Araujo et al., 2018[5]), blocking the inflammatory process, and, consequently, inflammatory disease progression.

Moreover, this ARB was able to restore redox homeostasis by upregulating the cellular antioxidant system, and consequently suppressing reactive oxygen species (ROS) generation and oxidative damage (Saber et al., 2019[100]). Olmesartan may inhibit the inflammation and oxidative stress induced by SAR-CoV-2 infection, reducing cell death and, consequently, the multiorgan failure observed in severe cases of COVID-19. This is supported by the results of a study by Kanamori et al. (2007[52]) that found that olmesartan downregulates the extrinsic and intrinsic apoptotic pathways by inhibiting caspase activation and reducing BAX and FAS expression. Therefore, olmesartan could inhibit the fulminant response by the immune system and suppress multiorgan failure, contributing to a reduction in the severity of the disease.

#### Telmisartan

Recently, Gurwitz (2020[37]) proposed the use of telmisartan as an alternative option for the management of COVID-19 patients prior to development of ARDS. This ARB produces marked suppression of inflammation, oxidative stress and apoptosis, leading to a decrease in cellular damage and multiorgan injury (Takeuchi et al., 2013[112]; Saber et al., 2019[99]; Zhang et al., 2019[142]).

Telmisartan is widely used for the treatment of patients with hypertension with concomitant diabetes mellitus (Wang et al., 2019[126]). This drug has a cardioprotective effect due to its capacity to inhibit inflammatory markers, myocardial apoptosis, and oxidative and endoplasmic reticulum stress, which results in an improvement in myocardial function (Sukumaran et al., 2011[111]). Moreover, telmisartan prevents pulmonary ischemia and reperfusion injury, decreases pulmonary vascular resistance and improves cough symptoms (Fan et al., 2016[27]), protecting the lung from oxidative and inflammatory injury. According to Zhang et al. (2019[142]) it reduces renal apoptosis and autophagy, ameliorating the renal impairment caused by chronic intermittent hypoxia.

Compelling evidence suggests that telmisartan possesses an anti-inflammatory and antioxidant profile (Takeuchi et al., 2013[112]; Choe et al., 2019[17]; Saber et al., 2019[99]). Saber et al. (2019[99]) showed that this ARB reduces IL1-β, TNF-α and IL-6 levels, and elevates IL-10 production, attenuating the inflammatory process by downregulating NFκB nuclear translocation/activation. Furthermore, telmisartan blocks myeloperoxidase activation and consequently reduces the infiltration of inflammatory cells into the injured organ (Saber et al., 2019[99]), mitigating oxide nitric production (Choe et al., 2019[17]). Additionally, telmisartan also stimulates the endogenous antioxidant system, reducing ROS formation (Saber et al., 2019[99]) and oxidative tissue damage. According to Takeuchi et al. (2013[112]) and Zhang et al. (2019[142]), this drug mitigates tissue injury by downregulating caspase pathway activation, reducing the number of apoptotic cells and death.

Table 1(Tab. 1) and 2(Tab. 2) summarize the main molecular and physiologic actions of telmisartan. Taken together, the evidence suggests that telmisartan may inhibit inflammation, oxidative stress and the apoptotic signaling pathway induced by SARS-CoV-2 infection, thereby reducing cell death and the multiorgan failure observed in the most severe cases of COVID-19. 

#### Valsartan

Valsartan is an angiotensin-II-receptor antagonist with specificity for the AT1R subtype and is commonly used for the treatment of cardiovascular disease. It demonstrates antihypertensive effects and slows the progression of chronic heart failure (Croom and Keating, 2004[20]). This ARB protects against cardiovascular, pulmonary and renal injury, mitigating organ failure through its ability to modulate the RAS system and inflammatory and oxidative mediators (Mohany et al., 2020[79]). Valsartan modulates the mRNA expression of ACE and AT1R (Li et al., 2016[62]), resulting in RAS inhibition and the prevention of renal and cardiac damage (Ulutas et al., 2021[121]). Moreover, studies have shown that valsartan attenuates pulmonary fibrosis in rats by blocking the NFκB signaling pathway and regulating the ratio of antifibrotic/profibrotic cytokines, resulting in an improvement in pathological changes in lung tissue (Mojiri-Forushani et al., 2018[80]). 

In this context, Mohany et al. (2020[79]) showed that rats treated with valsartan presented a substantial reduction in the levels of the pro-inflammatory cytokines TNF-α, IL-1β, and IL-6, and decreased activation of NF-kB. Moreover, the same authors reported that this drug restores antioxidant enzyme levels, indicating that the antioxidant effect of valsartan could mitigate cellular oxidative damage. Finally, some studies have reported that this ARB can modulate the expression of anti-apoptotic and pro-apoptotic proteins, decreasing caspase activation, and, consequently, cell death (Sakr et al., 2016[102]).

Table 1(Tab. 1) and 2(Tab. 2) summarize the main molecular and physiologic actions of valsartan, showing the main effects that could contribute to mitigating the effects of COVID-19, based on the significant anti-inflammatory and antioxidant profile of the drug. Valsartan could, therefore, block the “cytokine storm” and consequently suppress the fulminant response by the immune system and multiple organ failure, and be a promising possible treatment in the alleviation of COVID-19 symptoms. 

## Molecular Docking

**Figure 2:**
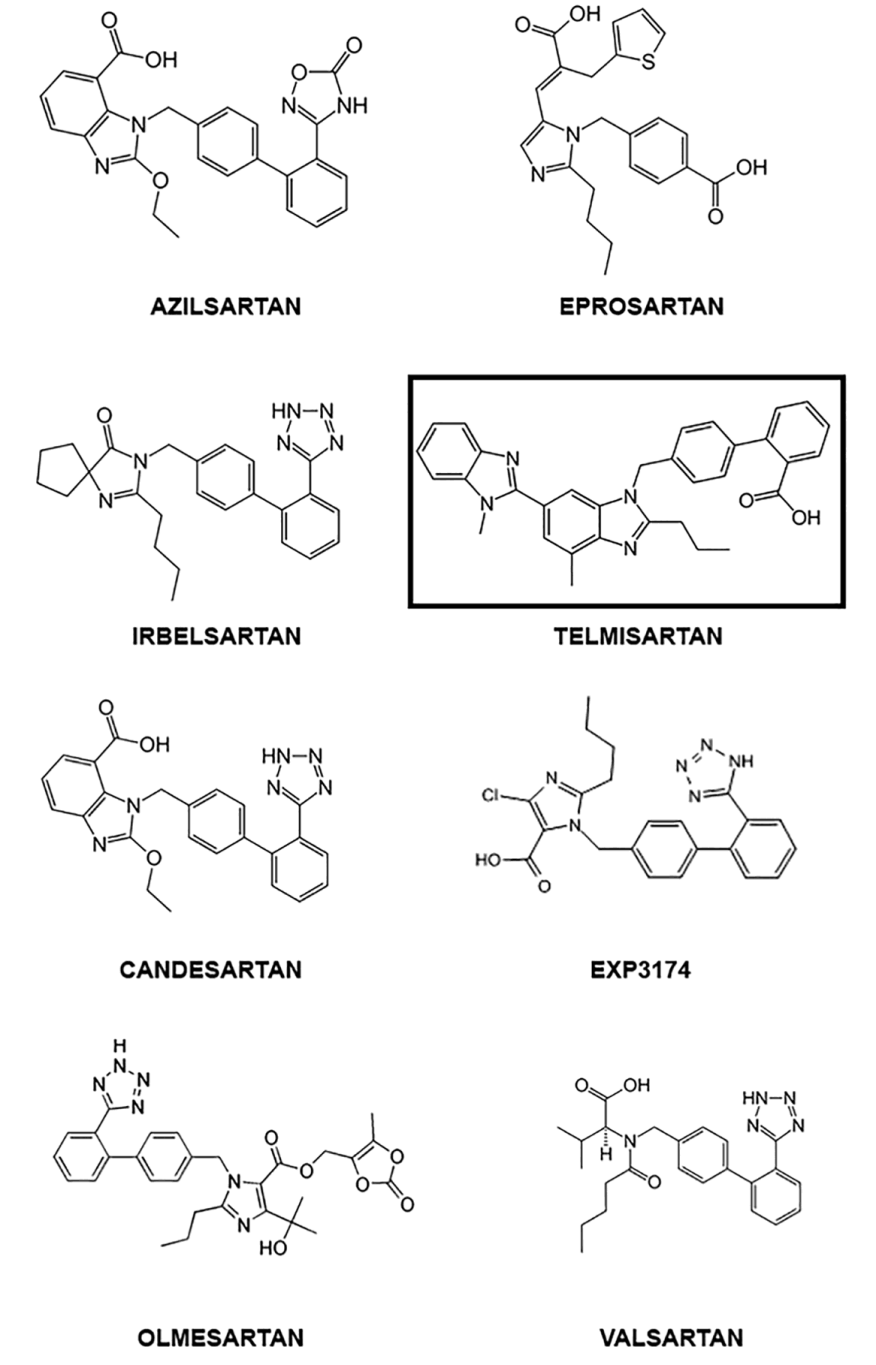
Chemical structures of AT1-inverse agonists

Computational molecular docking is a commonly used tool in drug repurposing studies and performs a structure-based computational analysis based on the binding efficiency predicted between a drug and its target molecule (Elfiky, 2020[25]). Molecular docking was performed to determine the binding efficiency between the ACE2-AT1-inverse agonists ligands and the AT1 receptor using the Molegro Virtual Docker v. 6.0.1 (MVD) (Thomsen and Christensen, 2006[118]). The structures of the receptors were downloaded from the Protein Data Bank (http://www.rcsb.org/pdb/home/home.do). The receptors investigated and respective ID PDB are ACE2AT1 - 6M17 (Yan et al., 2020[134]). For the analysis, all water compounds were deleted from the receptors and the default parameter settings were used with the same software: GRID of 15 Å of radius. The Moldock search algorithm was used, and the Moldock score [GRID] algorithm was used as the score function. The chemical structure of the AT1-inverse agonists studied are represented in Figure 2(Fig. 2).

Molecular docking predicted that all the AT1-inverse agonists tested possess high affinity with the AT1R. The AT1-inverse agonists exhibited binding energies for the AT1R between -133.224 (Valsartan) and -174.52 (Telmisartan) kcal/mol, with telmisartan> exp3174 > eprosartan > azilsartan > olmesartan > ibelsartan > candesartan > valsartan.

Interestingly, nitro functional groups in ARBs reinforce the nature of this group as one of the most important pharmacophorics for drugs which modulate this large family of receptors (Qin et al., 2009[95]). The presence of amide and ester groups in ARB drugs are responsible for their high affinity for the AT1R, and the effects they produce. Tetrazolate anions (nitrogen-rich five-membered heterocycles, a common pharmacophoric group of some ARB drugs) are more lipophilic than carboxylates, which promotes the passage of drug molecules through cell membranes, and makes them more resistant to metabolic degradation pathways, with a longer duration of action (Aziz et al., 2018[7]). The tetrazolate pharmacophoric group is common in AT1 inverse agonists (tetrazole ARB drugs) and the binding of the tetrazole moiety with the AT1R involves multiple binding through contact with residues of lysine and histamine that constitute the same subsite of the ligand binding pocket (Noda et al., 1995[86]).

The AT1R structures share the common architecture of having seven plasma membrane-spanning domains, or transmembrane domains (TMs) - which is why this family of proteins are known as 7TM receptors, connected to each other with three extracellular (ECL) and three intracellular loops (ICL), a disulfide bridge between ECL 2 and TM 3, and a cytoplasmic C-terminus containing an a-helix (Hx8) parallel to the cell membrane (Liapakis et al., 2012[63]). In the docking study, losartan (an AT1R), EXP3174 (the main losartan metabolite and a compound with inverse agonist activity) and olmesartan (an AT1 inverse agonist) clearly demonstrated their pharmacophoric binding with tetrazole and carboxyl groups (Matsoukas et al., 2013[68]). Interestingly, they demonstrated an additional interaction with Tyr87^2.63^ (a key side chain in the binding pocket of the secondary/tertiary structures at the seven-helical-bundle receptor domain) which was not contained in the pharmacophoric groups, so the hydroxyl group of Tyr87^2.63^ forms either a halogen bond interaction with the -Cl atom of losartan and EXP3174, or a hydrogen bond interaction with the hydroxyl group of olmesartan (Matsoukas et al., 2013[68]; Wilcken et al., 2013[129]). Here, EXP3174 and olmesartan demonstrated similar binding energy, but they have different affinities for the AT1R, and consequently different pharmacological efficacy that seems to benefit the modulation of the response mediated by this pathway (Bonde et al., 2010[11]). Takezako et al. (2015[114]) provided evidence of the essential role of the ECL2 (the second extracellular loop) residues Glu173 and Phe182 in the regulation of the conformational states of the AT1R, suggesting a potential strategy for developing new ARBs that directly target the ECL2, a key target for ligand binding and receptor activation. Moreover, the authors of the study reported that substitution of Val108^TM3^, Ala163^TM4^, Asn295^TM7^, and Phe182^ECL2^ in the constitution of the AT1R switched efficacy toward agonism for the ARBs in the activated state, but not in the ground state, although the link with ECL2 seems to be crucial. Furthermore, drugs such as azilsartan and eprosartan that are suggested to have greater inverse agonist activity in respect of the AT1R are not those with higher binding energies as suggested by our docking study, although the strong action on ECL2 seems to be one of the pillars of this activity when compared with binding energy (Zhang et al., 2015[140]; Takezako et al., 2018[115]).

**Figure 3:**
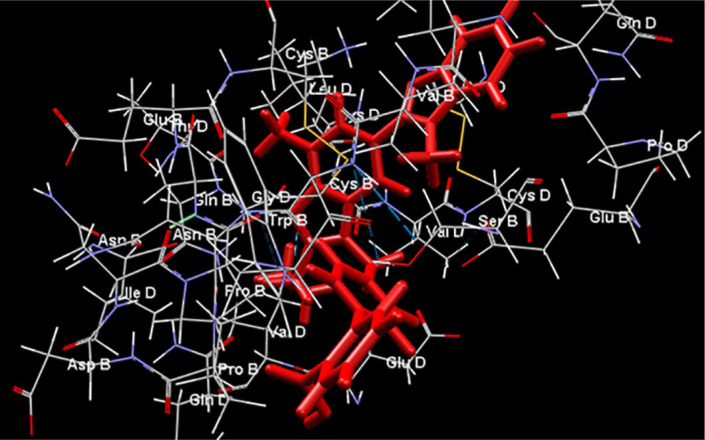
Telmisartan interactions in the binding cavity of the AT1 receptor from the docking study

Telmisartan had the strongest binding affinity to the AT1R in our docking study (Figure 3(Fig. 3)). However, eprosartan, an inverse agonist of the active state of the AT1R, despite having a lower binding energy, seems to be a better ARB drug because of its properties in respect of the ECL2 and receptor ligand binding pocket (Takezako et al., 2018[115]).

## Discussion and Conclusion

SARS-CoV-2 has affected millions of people worldwide; the disease caused by the virus is associated with inflammatory processes that can lead to severe pneumonia, cardiovascular failure, the dysfunction of several organs, and in severe cases can result in death (She et al., 2020[106]; Watkins, 2020[127]). 

As there is as yet no available specific and effective antiviral therapy to treat COVID-19 patients, a drug repurposing strategy is a rational response to the pandemic. In this context, among the wide range of anti-inflammatory and other drugs that should be considered, the AT1-inverse agonists present an interesting therapeutic option for COVID-19 treatment. Drugs with inverse agonism exhibit high efficacy and strong therapeutic effects for various disease states (Akazawa et al., 2009[2]), with actions that go beyond their antagonistic effects, giving them great potential as possible treatment options for SARS-CoV-2 infection.

AT1-inverse agonists exhibit anti-inflammatory (Saber et al., 2019[99]) and antioxidant (Saber et al., 2019[100]) profiles that could mitigate the hyperinflammation and oxidative stress caused by SARS-CoV-2 infection, thereby preventing disease progression and reducing the severity of cases and the number of hospitalizations. In addition, considering COVID-19 as a procoagulant disease, the effects of AT1-inverse agonists on the coagulation cascade could prevent thrombotic events and help to counteract the proinflammatory influence of cytokines. At this stage, it must be emphasized that current evidence suggests that AT1-inverse agonists also downregulate apoptotic protein expression or apoptotic pathway activation, reducing cellular death, and multiorgan failure. This hypothesis should be given serious consideration as the excessive inflammatory response commonly reported in severe cases of COVID-19 has been associated with a worsening of the respiratory condition. In addition, SARS-CoV-2 is capable of producing important *in vitro* cytopathic effects in human lung cell lines without significant cell death, even in the presence of a high concentration of viral particles, and is not proving to be a significant inducer of apoptosis in this condition (Chu et al., 2020[18]). This suggests that modulation of host systems, including RAS, during SARS-CoV-2 infection may play an important role in the pathogenesis of the disease, and be responsible for the intense focal lysis of type 2 pneumocytes. 

The RAS system has a possible central role in relation to COVID-19 because ACE2 is the main receptor for SARS-CoV-2, as well as for SARS-CoV. Although it is a marker of susceptibility to these coronavirus subtypes, its expression decreases markedly after coronavirus infection, which can generate excess Ang II in the tissue microenvironment with its pro-inflammatory, pro-thrombotic and pro-apoptotic effects, mainly produced by activating the AT1R. Interestingly, the previous use of exogenous Ang II (Giapreza®) in sepsis therapy showed complications similar to those found in severe cases of COVID-19, in addition to the intense vasoconstrictor phenomena that may explain the rapid change in clinical condition and poor clinical outcome, and its use as a vasopressor should be expressly avoided in patients with COVID-19 (Speth, 2020[109]). 

Currently, to the best of our knowledge, there are nine registered studies assessing the use of ARBs in treating patients with COVID-19, bringing promising results to this approach. As COVID-19 has a biphasic pattern, the effectiveness of therapy with ARBs could be related to the “timing” of infection, as well as patient risk factors, previous use of ARBs - even without important tachyphylaxis, and other class and molecular effects of a specific ARB. It is important to emphasize that at present the effect of ARB drugs on COVID-19 is a controversial subject in the literature, and several authors have shown that this kind of drug has a dual phase, with possible antagonistic effects (Dworakowska and Grossman, 2020[24]; Mehta et al., 2020[74]). Controlled studies are necessary to establish whether these drugs are effective in the treatment of COVID-19, and, if they are, what is the appropriate time to prescribe them. 

## Acknowledgements

The authors would like to thank FAPITEC-SE, CAPES, CNPq and EpiSERGIPE project. We dedicate this article to all the doctors and frontline health workers and other staff for their dedication in the fight against COVID-19. 

## Funding

No financial or otherwise, are declared by the authors.

## Conflict of interest

No conflicts of interest or otherwise, are declared by the authors.

## Authors' contributions

LH, MAS, JABF, ASB, FM, AASA, MTS, LS, PMF, LJQJ drafted manuscript LH, MAS, JABF, PMF, LJQJ edited and revised manuscript; LH, MAS, JABF, ASB, FM, AASA, MTS, LS, PMF, LJQJ approved final version of manuscript.

## Figures and Tables

**Table 1:**
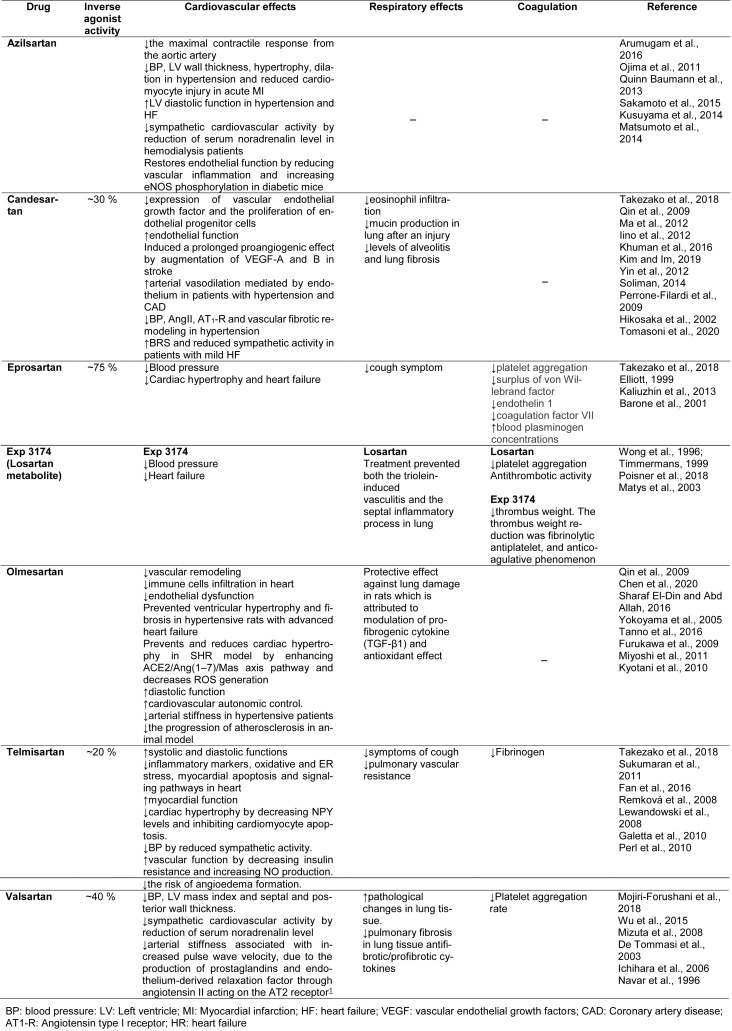
AT1 inverse agonist pharmacological effects

**Table 2:**
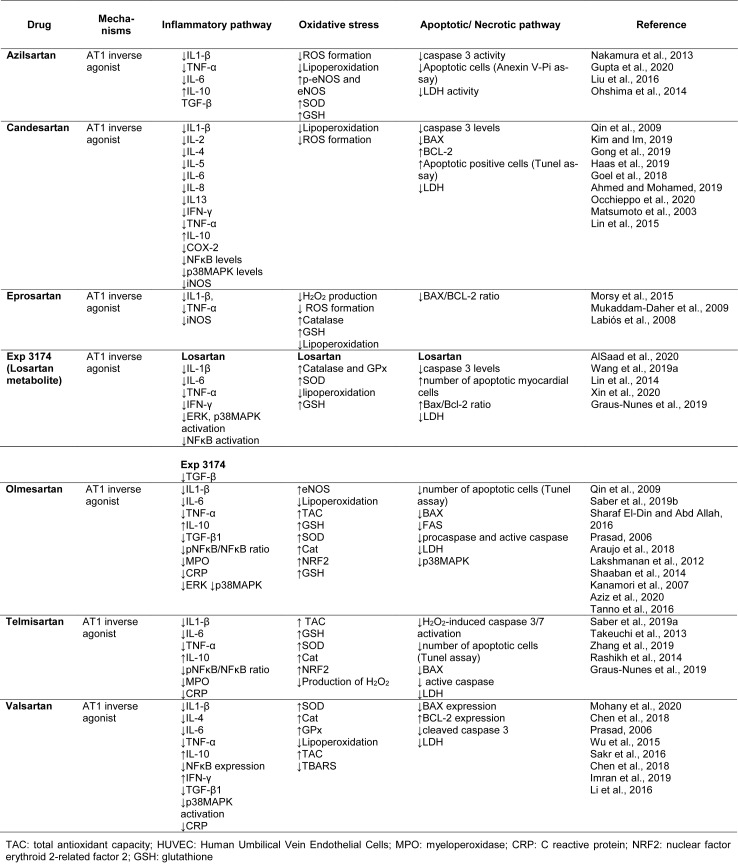
AT1 inverse agonist molecular mechanism
